# Cholinergic Modulation of Type 2 Immune Responses

**DOI:** 10.3389/fimmu.2017.01873

**Published:** 2017-12-19

**Authors:** Goele Bosmans, Gabriel Shimizu Bassi, Morgane Florens, Erika Gonzalez-Dominguez, Gianluca Matteoli, Guy E. Boeckxstaens

**Affiliations:** ^1^Translational Research Center for Gastrointestinal Disorders (TARGID), Department of Chronic Diseases, Metabolism and Ageing, KU Leuven, Leuven, Belgium

**Keywords:** parasympathetic nervous system, allergic inflammation, type 2 immunity, cholinergic anti-inflammatory pathway, neuroimmune interactions, vagus nerve

## Abstract

In recent years, the bidirectional relationship between the nervous and immune system has become increasingly clear, and its role in both homeostasis and inflammation has been well documented over the years. Since the introduction of the cholinergic anti-inflammatory pathway, there has been an increased interest in parasympathetic regulation of both innate and adaptive immune responses, including T helper 2 responses. Increasing evidence has been emerging suggesting a role for the parasympathetic nervous system in the pathophysiology of allergic diseases, including allergic rhinitis, asthma, food allergy, and atopic dermatitis. In this review, we will highlight the role of cholinergic modulation by both nicotinic and muscarinic receptors in several key aspects of the allergic inflammatory response, including barrier function, innate and adaptive immune responses, and effector cells responses. A better understanding of these cholinergic processes mediating key aspects of type 2 immune disorders might lead to novel therapeutic approaches to treat allergic diseases.

## Introduction

Evidence is accumulating that extrinsic nerves intensely and bidirectionally communicate with the immune system. Indeed, in 2000, Tracey and coworkers introduced the novel concept of the “inflammatory reflex,” where afferent vagal fibers sense peripheral inflammation and inform the brain on the inflammatory status to subsequently trigger vagal efferent fibers to dampen the inflammatory response (1). The cross talk between the vagus nerve and the immune system has been largely attributed to dampening of the innate immune system, in particular by reduction of cytokine release by splenic macrophages (2, 3). Since then, vagus nerve stimulation (VNS) has been evaluated in several inflammatory models characterized by not only activation of the innate immune system but also the adaptive immune system, particularly Th1 or Th17 immune responses (4, 5). Clinical observations, however, indirectly suggest that cholinergic immune modulation may also be of interest in atopic disorders characterized by T helper 2 (Th2) responses. Nicotine, a major constituent of cigarette smoke and a ligand for nicotinic acetylcholine receptors (nAChRs), has been implicated in anti-inflammatory effects seen in correlation with smoking. However, a rather complex relationship exists between smoking and the incidence of allergic diseases; on the one hand, cigarette smoke has been reported to cause impaired lung function, consequently aggravating asthma symptoms, and additionally also increase the risk to develop atopy, asthma, and food allergy (6–10). Oppositely, other reports indicated an inverse correlation between smoking and allergic disease incidence. Epidemiological studies demonstrated that the occurrence of asthma could be higher in former smokers, compared with active smokers (11–13). In addition, other studies have observed a negative association between smoking and the development of allergic sensitization (14, 15). In accordance with this, in a recent study Tilp et al. demonstrated that cigarette smoke reduces the allergen-induced Th2 response in the lung and the development of airway hyperreactivity (AHR) in a severe model of asthma in mice (16). Similarly, the adverse effects of excessive psychological stress on the incidence and severity of allergic disorders have been acknowledged for long time (17). Interestingly, anxiety and depression have been associated with a decrease in vagal tone, leading to the exploration of VNS as a treatment for depression (18–21). The possibility exists that the decrease in vagal tone leads to a reduction in cholinergic modulation promoting allergic inflammation. This theory is supported by a study demonstrating that impaired parasympathetic function increased susceptibility to inflammatory bowel disease in a mouse model of depression (22). Furthermore, acetylcholinesterase (AChE) inhibitors, which have been successfully used in animal models to activate the cholinergic anti-inflammatory pathway, are widely used in the clinic to treat Alzheimer’s disease (AD) (23–26). Curiously, AD patients treated with the AChE inhibitor, donepezil, showed increased serum levels of interleukin (IL)-4 and MCP-1, suggesting a possible shift toward a Th2 immune response (27). In addition, the effect of organophosphate compounds (OPCs), which are used as pesticides and are known AChE inhibitors, has been tested in allergic animal models. However, contrasting results have been obtained, whereas some studies mention an aggravation of allergic immune responses, other studies report an overall immunosuppressive effect (28–31). This discrepancy might be attributed to the timing of exposure, as a recent study showed that exposure to OPCs before allergen sensitization leads to worsening of allergic inflammation, while coinstantaneous exposure leads to suppression (31). Over the years, the importance of cholinergic modulation in inflammatory conditions has become increasingly clear. Although the role of parasympathetic regulation in allergic disorders has not been investigated extensively, more evidence is emerging supporting a role for cholinergic modulation in Th2-mediated disorders. Therefore, in this review, we aim to provide an overview of recent discoveries and its implications in relation to this research area.

## Pathophysiology of Allergic Inflammation

**Figure 1:**
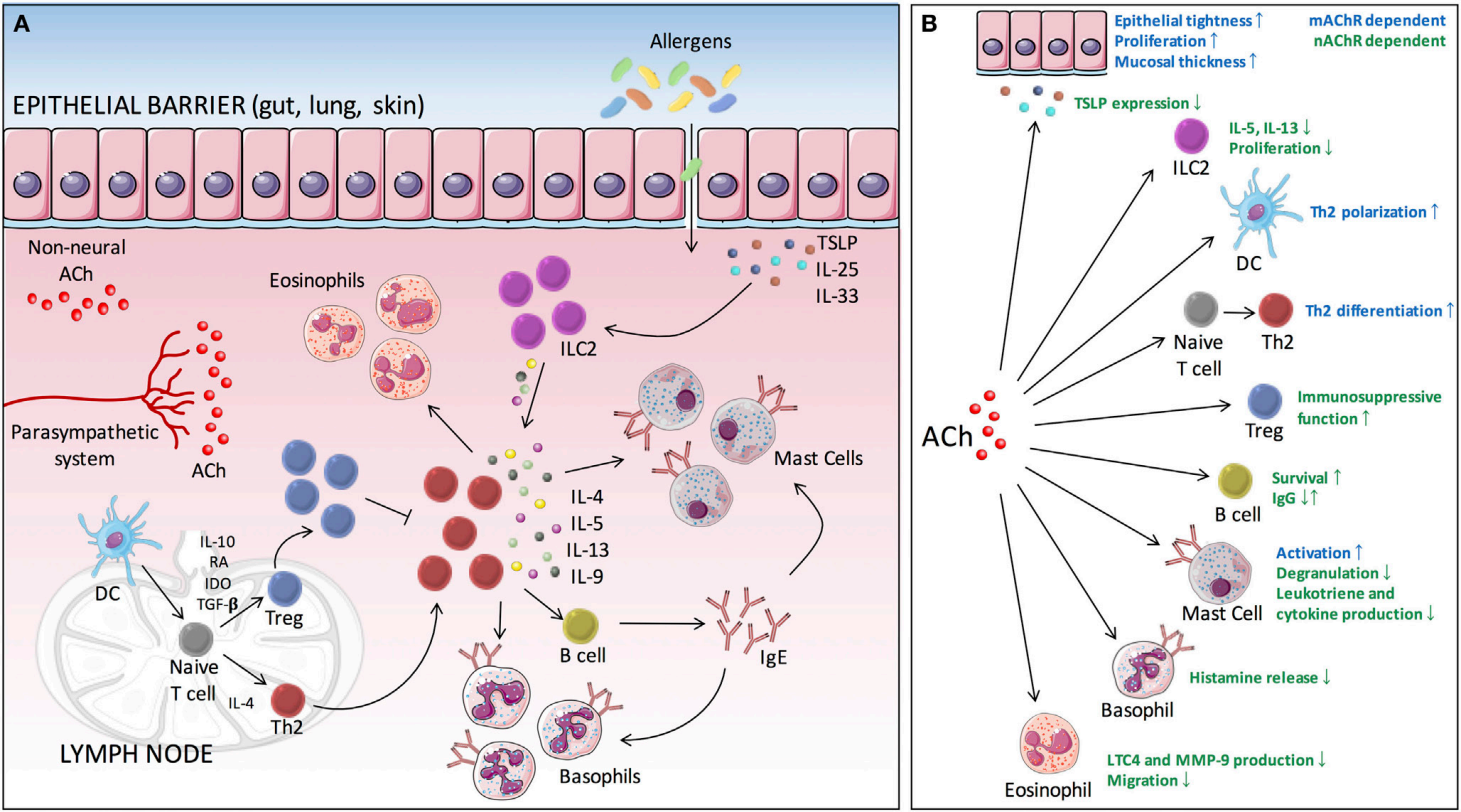
Cholinergic modulation of type 2-mediated inflammation. **(A)** Allergen exposure evokes the rapid release of epithelial cytokines (IL-25, IL-33, and TSLP), which in turn drives the production of type 2 cytokines by ILC2s. This creates an environment that further facilitates DCs to promote the differentiation of naive T cells toward Th2 cells. These Th2 cells will then orchestrate different events of the inflammatory allergic response, through the release of type 2 cytokines (IL-4, IL-5, IL-9, and IL-13), including the attraction of effector cells (eosinophils, mast cells, and basophils) to the site of inflammation. In addition, B-cell class switching is promoted, leading to the production of allergen-specific IgE. Binding of antigen to IgE bound on mast cells and basophils leads to IgE cross-linking and subsequent activation. **(B)** ACh is able to modulate several key processes of the allergic inflammatory response *via* muscarinic and nicotinic receptors. ACh, acetylcholine; TSLP, thymic stromal lymphopoietin; DC, dendritic cell; ILC2, type 2 innate lymphoid cell; Th, T helper cell; IgE, immunoglobulin E; mAChR, muscarinic acetylcholine receptor; nAChR, nicotinic acetylcholine receptor; IL, interleukin; Th2, T helper 2.

At mucosal surfaces, the epithelium is crucial for maintaining a physical barrier and protect against environmental insults and allergens. In addition, it forms a functional barrier allowing communication between the internal and external environment. A whole array of different immune cells exists within the mucosal barrier, constantly surveying the environment and ready to provide innate and adaptive immunity. Many allergic diseases typically coincide with disorders of the epithelial barrier, which is associated with loss of epithelial differentiation, reduced junctional integrity, and impaired innate defense. For a long time, barrier dysfunction was seen as a consequence of the aberrant immunological response toward allergens. Recent evidence, however, suggests that barrier dysfunction also may act as an important initiator and player in the pathophysiology of allergic inflammation. The barrier can be disrupted by a number of factors arising from the external environment, such as proteases, toxins, or injury, but also endogenous factors such as hormones, diet, and circadian clock could contribute. Disruption of the barrier might result in increased antigen uptake and can drive predisposition to allergic sensitization (32, 33). On exposure, allergens are sensed by epithelial cells, which in turn lead to the rapid release of epithelial cytokines, IL-25, IL-33, and thymic stromal lymphopoietin (TSLP) (34). These epithelial cytokines are able to induce expression and release of type 2 cytokines by type 2 innate lymphoid cells (ILC2s). This creates an environment that promotes the generation of allergen-specific Th2 cells, a process that is mainly driven by antigen presenting cells, primarily dendritic cells (DCs). DCs are responsible for taking up, processing and presenting allergens to naïve T cells. Th2 cells then play a central role, orchestrating different events of the inflammatory allergic response, mainly through the release of type 2 cytokines such as IL-4, IL-5, IL-9, and IL-13. These events include the attraction of effector cells such as eosinophils, mast cells, and basophils to the site of inflammation. In addition, B-cell class switching will be promoted under the influence of IL-4 and IL-13, leading to an increased production of allergen-specific immunoglobulin E (IgE). Upon allergen reexposure, IgE is able to bind FcεRIα on the surface of mast cells and basophils leading to their activation. This cascade of events can occur at different sites throughout the body and represent the underlying mechanism of disorders such as allergic rhinitis, asthma, food allergy, and atopic dermatitis (Figure 1) (35, 36).

## The Cholinergic Nervous System in Gut, Lung and Skin

Barrier surfaces such as the gastrointestinal (GI) tract, respiratory tract, and skin are densely populated by neurons and immune cells that constantly sense and respond to environmental challenges, including allergens. The peripheral nervous system (PNS) consists of the somatic nervous system and the autonomic nervous system. The latter can be further subdivided into the parasympathetic, sympathetic, and enteric nervous system (ENS). The different neurons of the PNS have been found to communicate with the immune system through the release of neuromediators from their nerve terminals. The parasympathetic nervous system primarily uses the neurotransmitter acetylcholine (ACh). As in this review, the focus will be on cholinergic modulation of the immune response, we will first describe the parasympathetic innervation and cholinergic input at the different epithelial barriers typically involved in allergic conditions.

The gut is densely innervated by the autonomic nervous system, consisting of the extrinsic innervation and the ENS, located within the intestine. The vagus nerve, providing a bidirectional connection between the brain and the gut, represents the main extrinsic parasympathetic nerve in the GI tract, where it mainly controls secretion, vascularization, and gastrointestinal motility. Preganglionic efferent vagal nerve fibers extensively innervate the GI tract, displaying the highest density in the stomach and further decreasing in the small bowel and colon, and establishing connections with postganglionic neurons primarily located in the myenteric plexus (37, 38). However, as vagal efferents only synapse with cholinergic enteric neurons in the myenteric plexus, it is likely that they affect mucosal immune responses indirectly through activation of cholinergic ENS neurons releasing ACh (39).

In the lung, the parasympathetic nervous system plays a prominent role in the control of airway smooth muscle tone. ACh released from postganglionic neurons induces bronchoconstriction, mucus secretion, and bronchial vasodilation, primarily mediated *via* binding on muscarinic receptor M3 (40, 41). For this reason, anticholinergic and muscarinic antagonists have been used to treat bronchoconstriction in asthma. The prominent role of the parasympathetic nervous system in the pathophysiology of asthma makes it challenging to investigate its role in the modulation of the immune response.

In contrast to the GI and the respiratory tract, the skin is devoid of parasympathetic innervation (41). This might question a role for cholinergic modulation of immune responses in the skin and in diseases, such as atopic dermatitis. However, the skin contains other sources of ACh, in particular keratinocytes (42), but in fact almost every cell, including epithelial, endothelial, and immune cells can produce ACh. Hence, this so-called “non-neural cholinergic system” might not only be of relevance in the skin but also in the gut and lung (43).

## Cholinergic Modulation of Barrier Function

Improving epithelial barrier function could result into a decreased access of allergens, limiting the subsequent type 2 inflammatory response. Although there is no direct evidence for cholinergic modulation of epithelial barrier function in allergic disorders, some studies do suggest a role for ACh in modulating barrier integrity. ACh was shown to play a role in the regulation of epithelial tightness in pig colon cultures. Incubation with carbachol resulted into an increased transepithelial electrical resistance, an effect that was inhibited by atropine, suggesting involvement of muscarinic acetylcholine receptors (mAChRs) (44). In addition, muscarinic agonists where shown to stimulate epithelial cell proliferation, increasing mucosal thickness in the intestine. Moreover, in several inflammatory conditions, cholinergic modulation was seen to protect barrier integrity due to improved tight junction protein expression (45–48). However, this effect is probably indirectly regulated by the downregulation of pro-inflammatory cytokines. Although the cholinergic modulation of barrier function in type 2-mediated diseases has been relatively unexplored so far, it might hold yet undiscovered potential toward therapeutic interventions.

The epithelium should not be considered as merely a physical barrier controlling the uptake and transport of antigens; in addition, it should be seen as an active contributor to the mucosal environment helping to shape local immune reactions. Epithelial derived cytokines IL-25, IL-33, and TSLP have been shown to play a role in the initiation of type 2 allergic responses. Preventing the expression and release of epithelial cytokines might be sufficient to prevent the subsequent Th2 immune response. Evidence that cholinergic modulation might influence this process was recently provided by a study showing inhibition of TSLP expression both *in vitro* and *in vivo* by nicotine. This effect was abolished by pretreatment with α7 nAChR antagonists, showing the involvement of α7 nAChR (49).

Experimental and clinical data have supported a role for type 2 cytokines in the regulation of intestinal barrier function. IL-4 and IL-13 have been shown to increase intestinal permeability, an effect that is mediated *via* phosphoinositide 3-kinases (50–53). In addition, allergic effector cells such as mast cells and eosinophils release powerful inflammatory mediators, such as proteases and cytokines, further contributing to barrier dysfunction (52, 54–56). Cholinergic modulation of immune cells involved in the allergic inflammatory response might therefore, in addition to direct effects that will be described in detail below, also contribute to effects on barrier function.

## Cholinergic Modulation of the Type 2 Innate Immune Response

The innate immune system plays an important role in the initiation of subsequent adaptive immune response. Key players of type 2 innate immunity include recently described ILC2s that produce cytokines promoting Th2 differentiation by DCs, which are considered as a crucial link between innate and adaptive immunity.

Interestingly, a recent study showed that ILC2 might be modulated by cholinergic signals. Indeed, α7 nAChR expression has been shown on ILC2, whereas *in vitro* incubation of pulmonary ILC2s with GTS-21, a specific α7 nAChR agonist suppressed production of IL-5 and IL-13 cytokines, proliferation, and GATA3 expression. In addition, GTS-21 treatment ameliorated ILC2 induced AHR. Similar observations were made in a humanized ILC2 AHR mice model (57). These findings suggest that cholinergic modulation of ILC2s *via* the α7 nAChR is a potential target for therapeutic intervention in type 2-mediated disorders.

In contrast to the well described anti-inflammatory effect of ACh on macrophages (39, 58–60), the effect of cholinergic modulation on DCs has been far less studied. Aicher et al. demonstrated the expression of α7 nAChR on human monocyte-derived DCs, which was upregulated in response to its ligand nicotine (61). In addition, murine bone marrow-derived DCs expressed mRNAs encoding the five mAChR subtypes (M1–M5) and the α2, α5, α6, α7, α10, and β2 nAChR subunits (62). Earlier reports suggested an anti-inflammatory effect of nicotine reducing endocytosis, phagocytosis, the production of pro-inflammatory cytokines IL-12, IL-1β, and TNF-α and the ability to induce a Th1 response (63). By contrast, other reports stated that nicotine activated DC function, enhancing endocytosis, stimulation of T cell proliferation, and the production of IL-12 (61, 64). These opposing results might be due to differences in maturation and activation state. More recently, Gori et al. demonstrated a role for ACh in polarizing DCs toward a Th2-promoting phenotype. Treatment of DC with ACh stimulated the expression of the Th2-promoter OX40L, the production of Th2-chemokines CCL22 and CCL17, and the synthesis of IL-4, IL-5, and IL-13 by T cells (65). This study was in line with earlier findings by Liu et al. showing that the muscarinic receptor agonist, methacholine, increased OX40L in DCs (66). These data suggest an involvement of mAChRs in the Th2 polarizing effect of ACh on DCs.

## Cholinergic Modulation of the Type 2 Adaptive Immune Response

Given that DCs form a bridge between the innate and adaptive immune responses, cholinergic modulation of these cells as described earlier will definitely influence consequent adaptive immune responses. However, both T and B lymphocytes are also directly modulated by ACh, as several nAChR and mAChRs subtypes have been reported in both cell types (67–70).

As for other immune cells, also T cells show a high degree of plasticity with respect to their cholinergic receptor network. Depending on their activation state, differentiation state or even environmental cues, T cells will express a different repertoire of ACh receptors (71). So far, especially α7 nAChR has been investigated in the context of the cholinergic anti-inflammatory pathway (1). Although the expression of the α7 nAChR subunit has been demonstrated in activated CD4 T cells, surprisingly Th1, Th2, or Th17 cells do not express detectable levels of this receptor (71). In addition, Although vagotomy has been shown to increase T cell proliferation and production of pro-inflammatory cytokines, this effect is not attenuated by selective α7 nAChR agonists, suggesting the involvement of other subunits such as α4, β2, and α5 (69, 72, 73). Other studies, however, do highlight the importance of the α7 nAChR in the anti-inflammatory effect of nicotine. Interestingly, a study by Galitovskiy et al. showed that α7 nAChR mRNA expression in colonic CD4 T cells was highly dependent on the inflammatory milieu. IL-4 was shown to upregulate α7 nAChR gene expression, whereas it was reduced by IL-12, possibly explaining the dualistic effect of nicotine in ulcerative colitis (Th2) versus Crohn’s disease (Th1/Th17) (74). This finding is especially of interest in allergic disorders, which are characterized by a strong type 2 inflammatory environment. In addition, a more recent study proposed α7 nAChR as a critical regulator for the immunosuppressive function of Tregs, mainly due to upregulation of immunoregulatory molecules CTLA-4 and FoxP3, and decreasing IL-2 levels (75). Considering the crucial role of Tregs in immune homeostasis and their capacity to suppress Th2 responses to allergens and other type 2 effector cells, novel ways to promote Treg cell stability and function, are being explored extensively in the treatment for allergic diseases. Whereas there has been an increasing appreciation for nicotinic receptors in the modulation of inflammation and homeostasis, muscarinic receptors have received much less attention so far. Of note, several studies have highlighted the importance of the M3 receptor in the generation of type 2 immune responses. Stimulation of mAChRs on naïve CD4 T cells promotes Th2 and Th17 lineages, while blockade of mAChR will lead to Th1 differentiation (71). Moreover, two recent studies demonstrated the importance of M3 receptor activity in the generation of Th2 responses in an enteric nematode infection model and an allergic airway inflammation model (76, 77). These studies suggest that the M3 receptor opposes the effect of α7 nAChR receptor activation (77). It should be emphasized though that the range of doses that activates each receptor is some log units apart, questioning if this interplay is indeed of physiological relevance. So far, most findings concerning cholinergic modulation of T cells are based on *in vitro* studies, hardly representing the complex nature of the inflammatory environment. Therefore, to better understand the functional consequences of cholinergic activation on T cells *in vivo*, further studies in experimental models are needed.

As mentioned earlier, functional expression of nAChR subunits and mAChRs subtypes has also been demonstrated in B cells (67–70). Information on the functional implications of cholinergic receptor activation is, however, rather limited. Nicotine affects the survival of B lymphocyte precursors during differentiation, an effect that was dependent on both α7 and β2 subunits (78). In addition, effects on antibody responses have been reported. A study by Skok et al. described a role α4β2 and α7 nAChRs in the formation of the pre-immune status and the regulation of antibody response, possibly by affecting CD40 expression and/or signaling. Mice lacking α4, β2, or α7 nAChR subunits exhibited lower levels of IgG antibodies (79, 80). By contrast, Fuji et al. reported higher levels of IgG1 antibodies in α7 nAChR knockout mice (81). The reason for this discrepancy is still unclear. However, both studies reported increased levels of IgG antibodies after immunization in the absence of α4, β2, or α7 nAChR subunits. Of note, while the effect on antibody response could be directly regulated *via* cholinergic receptors on B cells, it could also be attributed to cholinergic inhibition of pro-inflammatory cytokines by other immune cells, indirectly leading to the suppression of IgG antibodies. Also in humans, effects on antibody responses have been observed. When comparing smokers with non-smokers, the former were shown to exhibit lower levels of all serum immunoglobulins, except for IgE (82, 83). Unfortunately, cholinergic modulation of B cells in inflammatory conditions has been relatively unexplored until now, so its role in allergic inflammation remains unclear.

## Cholinergic Modulation of Allergic Effector Cells

Mast cells, basophils, and eosinophils are considered as central effector cells in allergic inflammation. Their activation and subsequent release of granule stored mediators leads to many of the characteristic pathophysiological features observed in allergic responses. Ways to modulate these effector cells and to suppress their activation hold promise for the development of therapeutics for allergic diseases. All three types of granulocytes are shown to express cholinergic receptors, and are thus prone to cholinergic modulation.

Both muscarinic and nicotinic cholinergic receptors have been identified on mast cells (84–86). Moreover, mast cell–nerve associations have been shown in both small intestine and colon (87–89). Vagotomy in rats resulted into a decrease of mast cells in the jejunal mucosa (90). VNS on the other hand was shown to elevate histamine levels in the jejunal wall. To what extent this effect was due to increased synthesis or decreased release of histamine remains unclear (91). Thus far, studies describing the functional effects of cholinergic mast cell modulation have been rather conflicting. Early studies reported activation rather than inhibition of mast cell degranulation by ACh *via* muscarinic receptors (92–94). Other studies, however, failed to show an effect of ACh on mast cell degranulation (95, 96). This discrepancy was addressed by Masini et al., demonstrating that incubation with IgE increased the sensitivity of mast cells to ACh and induced a more homogeneous response (94). Nicotinic receptors on the other hand seem to mediate an anti-inflammatory effect. Expression of nicotinic receptor subunits α4, α7, and β2 was found in mouse bone marrow-derived mast cells. In addition, nicotine was shown to inhibit antigen-IgE-induced degranulation of mast cells in a dose dependent manner, an effect that was mimicked by the α7 nAChR subunit agonist, GTS-21. α-Bungarotoxin, a specific α7 antagonist, significantly inhibited the suppressive effect of GTS-21, further confirming the observed effect is α7 nAChR dependent (85). In line, α7, α9, and α10 receptor subunits are expressed in a rat mast cell/basophil cell line, RBL-2H3, in which nicotine induces suppression. Although nicotine did not inhibit degranulation, it suppressed late phase FcεRI-induced leukotriene and cytokine production, an effect that could be abrogated by siRNA mediated ablation of α7, α9, or α10 nAChRs (97). Interestingly, a recent study by Yamamoto et al. demonstrated a potential therapeutic effect for the cholinergic anti-inflammatory pathway in an experimental murine model of food allergy. Vagal stimulation by using 2-deoxy-d-glucose, a central vagal stimulant, ameliorated the development of food allergy, and was reversed by the nAChR antagonist, hexamethonium. Moreover, similar effects were obtained with nicotine and GTS-21, indicating the involvement of α7 nAChR. It was suggested that the amelioration of allergic symptoms where due to the suppression of mucosal mast cells; however, no direct evidence was provided. The possible involvement of other immune cells can thus not be excluded (89).

Basophils share many similarities with mast cells and are often considered as their circulating counterparts. However, the cholinergic modulation of basophils has been far less investigated compared with mast cells. So far, the earlier mentioned rat mast cell/basophil cell line, RBL-2H3 was shown to express α7, α9, and α10 nAChR receptor subunits (97). In addition, fluorescently labeled α-bungarotoxin was shown to bind to the surface of a human basophil cell line, and more recently the presence of nAChR α4, α7, and α1/α3/α5 subunits was shown in human blood basophils by flow cytometry (98, 99). In parallel with the observations in mast cells, Thompson-Cree et al. showed that nicotine agonists were able to inhibit IgE-induced histamine release from human peripheral blood basophils (100). In addition, a recent study showed similar *in vitro* and *in vivo* inhibition of human blood basophils from mild allergic asthma patients using ASM-024, a novel compound with nicotinic and muscarinic effects (99). The anti-inflammatory effect of ASM-024 was moreover confirmed in a murine model of allergic airway inflammation (101).

Eosinophil–cholinergic nerve interactions have been documented in several tissue compartments under inflammatory conditions, including lung, intestine, nose, and skin (102–108). The interaction between eosinophils and nerves seems to be mediated *via* adhesion molecules ICAM-1 and VCAM-1 and has been correlated with cholinergic nerve remodeling (103, 109). Eosinophil adhesion results in degranulation leading to the release of mediators such as major basic protein (MBP) and eosinophil peroxidase (EPO) (103). Binding of MBP and EPO leads to increased release of ACh due to upregulation of choline acetyltransferase and vesicular ACh transporter gene expression, and in addition due to antagonistic effect on inhibitory M2 receptors (110–112). Recently, modulation of cholinergic nerves by eosinophils has become increasingly clear and might contribute to pathological processes, such as smooth muscle contraction and increased mucus production, observed in allergic conditions. However, a possible anti-inflammatory effect of ACh on eosinophils should not be discarded. Expression of α3, α4, and α7 nAChR subunit transcripts and protein has been demonstrated in human blood cells. Moreover, it was shown that stimulation of these cells *in vitro* with DMPP, a nicotinic agonist, downregulated eosinophil function. DMPP inhibited LTC4 production, eosinophil migration, MMP-9 production, and intracellular calcium mobilization (113). In addition, DMPP was used earlier in an *in vivo* model of asthma, where it prevented airway hyperresponsiveness and reduced the number of lymphocytes and eosinophils. The obtained results could be in part attributed to the observed effects on eosinophil function; however, additional effects on other immune cells cannot be excluded (113, 114).

## Conclusion

In recent years substantial advances have been made with respect to understanding the complex interactions between the nervous system and the immune system. The findings described in this review have highlighted a so far underappreciated role for the parasympathetic nervous system and cholinergic modulation in controlling type 2 adaptive immune responses. Several key players of the pathophysiological mechanism underlying allergic disorders are subject to cholinergic modulation (Figure 1) and could thus be exploited in the search for new therapeutic approaches to treat allergies. However, it should be noted that neuroimmune modulation is not only limited to the parasympathetic nervous system. Different neurotransmitters, originating from the parasympathetic, sympathetic, and ENS, are able to influence each other. For instance, neurotransmitters of the parasympathetic and the sympathetic nervous system are often found to exert antagonistic effects. In addition, immune cells render an enormous plasticity what expression of neurotransmitter receptors is concerned. A number of factors including the local microenvironment, which will differ in health versus disease, the maturation or activation state of cells, will influence receptor expression. Predicting the outcome of therapeutic interventions using specific receptor agonists and antagonists will therefore be challenging. A deeper understanding of how the different branches of the nervous system interact with each other and with the immune system, in both homeostasis and inflammation, will hold considerable promise for the development of new and innovative therapeutic strategies for allergic disorders and other inflammatory conditions.

## Author Contributions

Drafting the manuscript: GB and GEB; revising the manuscript critically for important intellectual content: GEB, GSB, MF, EG-D, and GM.

## Conflict of Interest Statement

The authors declare that the research was conducted in the absence of any commercial or financial relationships that could be construed as a potential conflict of interest.
